# Noise-Adaptive Non-Blind Image Deblurring

**DOI:** 10.3390/s22186923

**Published:** 2022-09-13

**Authors:** Michael Slutsky

**Affiliations:** GM Technical Center Israel—R&D Lab, 13 Arie Shenkar St, Herzliya 4672513, Israel; michael.slutsky@gm.com

**Keywords:** convolutional neural networks, image restoration, non-blind deconvolution, regularization parameter

## Abstract

This work addresses the problem of non-blind image deblurring for arbitrary input noise. The problem arises in the context of sensors with strong chromatic aberrations, as well as in standard cameras, in low-light and high-speed scenarios. A short description of two common classical approaches to regularized image deconvolution is provided, and common issues arising in this context are described. It is shown how a pre-deconvolved deep neural network (DNN) based image enhancement can be improved by joint optimization of regularization parameters and network weights. Furthermore, a two-step approach to deblurring based on two DNNs is proposed, with the first network estimating deconvolution regularization parameters, and the second one performing image enhancement and residual artifact removal. For the first network, a novel RegParamNet architecture is introduced and its performance is examined for both direct and indirect regularization parameter estimation. The system is shown to operate well for input noise in a three orders of magnitude range (0.01–10.0) and a wide spectrum of 1D or 2D Gaussian blur kernels, well outside the scope of most previously explored image blur and noise degrees. The proposed method is found to significantly outperform several leading state-of-the-art approaches.

## 1. Introduction

Video cameras are the most common sensing modality in modern vehicles. The number of cameras per vehicle is constantly growing, both in the retail sector, e.g., in Advanced Driver Assistance Systems (ADAS), and in the Autonomous Vehicle (AV) domain. There is a constant demand to reduce the cost of imaging systems without compromising the quality.

Raw images acquired by digital image sensors pass through a rather long image signal processing (ISP) chain [1] before they are handed over to a perception system for object detection, recognition, tracking, or a variety of other generic or domain-specific tasks [2]. Success of perception systems critically depends on image quality [3], and is therefore constantly pushing the requirements for resolution, frame rate, dynamic range etc. For the last decade or so, we have witnessed a staggering improvement in image quality of mobile phone and automotive-grade cameras, often comparable to image quality of professional-grade cameras. This improvement came mainly from the direction of ISP modules, driven by rapid progress in image processing algorithms, which in turn was possible due to a significant increase in the available processing hardware. Many such algorithms were invented to compensate for artifacts introduced by relatively cheap optics (e.g., aberrations and distortions) or small pixel size (e.g., noise and cross-talk).

In this work, the problem of image blur is addressed. This is a rather old and cross-disciplinary topic—people have been dealing with blurry images more or less since the invention of photography, in most imaging domains and at all scales—from astronomy [4] to microscopy [5]. Typically, image blur is motion-induced [6] or caused by optical aberrations [7,8]. For instance, maybe the most well-known imaging system optimization challenge deals with choosing a correct value for the exposure time. If it is too short, the image appears very noisy, if it is too long—camera motion or intrinsic motions in the scene smear the image and it becomes too blurry. Thus, long-range cameras designed for highway usage and high-speed driving tend to produce noisy images in low-light conditions, whereas short-range surround view cameras are more lenient when it comes to exposure times. In any case, to achieve less noise means using more expensive sensors or longer exposure times, which means image blur.

Until the introduction of digital imaging more than half a century ago, probably the only way to deal with image blur was to avoid it by using high-grade optics and sensitive photographic film. Digital imaging allows us to build image formation models, to isolate degradation stages, and to formulate image restoration as an inverse numerical problem [9]. As the following sections show, such formulation often paves way to using well-known techniques from other, more established fields. The first publications on digital image deblurring appeared in the late 60’s and early 70’s [10,11,12]. Since then, image deblurring has flourished into a rather wide field that can be roughly divided into two main sub-fields. The first kind of deblurring problems are referred to as “non-blind deblurring”. In this class of problems it is assumed that all components of image formation model are known. In particular, the blur kernel (often referred to as the point-spread function, or PSF) is assumed to be either directly measured in some calibration procedure or inferred from auxiliary measurements, e.g., from motion sensors readings [13]. The second class of problems is called “blind deblurring”, which means that the PSF is not known. As often happens, there is also a rather dense spectrum of problems that fall somewhere in between the two classes. For instance, the exact shape of the PSF may not be known, but assumptions can be made regarding its origin, extent, regularity etc. To appreciate the vast progress in the field up until 2014, the reader is referred to a comprehensive review article by Wang and Tao [14].

Naturally, blind deblurring problems are more challenging than non-blind deblurring ones. For many approaches, the latter can be considered as a central block in a generic solution flow for blind deblurring: find a deblurred image given a PSF, estimate the error, refine the PSF etc. Still, as the next sections show, even if one is fortunate enough to know the PSF, finding a reliable solution is a rather non-trivial task. In particular, the solution should be properly regularized to be of any use, which requires inference of regularization parameters. The latter, in turn, are strongly influenced by the level of noise in the blurred image as well as by intricate texture statistics of the image. Often, regularized solutions exhibit common artifacts, such as boundary artifacts or Gibbs phenomena that inevitably accompany most solutions to ill-posed inverse problems. Much effort has been invested in fighting these artifacts, either as a post-processing step or by devising specialized boundary conditions and inverse operators.

As with almost every field in image processing and computer vision, application of deep learning techniques to image deblurring during the last decade, generated a great number of impressive results [15,16]. Most researchers are interested in blind deblurring since this is where most challenges are. Although there was quite impressive progress on that front [17,18,19], existing deep neural networks (DNNs) are able to learn PSFs of a rather limited extent, so that the problem of blind deblurring remains largely unsolved. Non-blind deblurring has benefited from deep learning as well. For instance, there has been much activity around designing suitable architectures and ways to incorporate known PSF kernels into them [20,21,22]. Another approach proposed by Xu et al. [23] is to use classical deconvolution followed by a DNN trained to remove residual deconvolution artifacts. This approach was further explored by Wang and Tao [24], who proposed using very deep architectures to predict residuals between a pre-deconvolved image and the corresponding sharp image. It is argued that this approach is noise-blind in the sense that it is able to handle different blur kernels and various noise levels. However, in both cases it is assumed that the pre-deconvolved image actually exists, which implies knowledge of suitable regularization parameters, or at least, knowledge of input noise [9].

This work addresses the problem of non-blind image deblurring for almost arbitrary levels of input noise. The two-step approach is generalized to the case of arbitrary noise in a three orders of magnitude range (10${}^{-2}$–10.0) and arbitrary 1D or 2D blur kernels.

This paper is organized as follows. First, a short review of classical approaches to regularized image deconvolution is provided, and common issues arising in this context are described. Then it is demonstrated how a two-step DNN based deblurring works, and how it can be improved by treating regularization parameters as trainable neural network weights. Next, the two-step approach is generalized by introducing RegParamNet, a 1D convolutional network that estimates optimal regularization parameters for a given blurred and noisy input. Finally, the results of the generalized approach are analyzed and possible extensions are discussed.

## 2. Regularized Deconvolution

### 2.1. Tikhonov Regularization

In this work it is assumed that the blurred image $J_{B}$ is related to the original image J through a linear convolution with additive noise:

(1) $$J_{B}=K*J+\Xi ,$$

where K is the blur kernel and Ξ is a random Gaussian noise matrix. It is also assumed that K is known. To avoid noise amplification, Tikhonov [25] suggested to reformulate (1) as a regularized least-squares (LS) problem, such that

(2) $$J_{\lambda }^{\left(LS\right)}=\mathrm{argmin}\left\{{∥K*J-J_{B}∥}^{2}+\lambda^{2}{∥L*J∥}^{2}\right\}$$

Here, L is a linear operator (usually, an identity operator or a derivative) that is chosen in accordance with the property of the solution that needs to be regularized. For 1D blur, the convolution operation can be expressed as multiplication of the image J by a blur matrix. For instance, the solution for horizontal blur is

(3) $$J_{\lambda }^{\left(LS\right)}=J_{B}K^{T}{\left(K^{T}K+\lambda^{2}L^{T}L\right)}^{-1}$$

Throughout this work, the identity operator L=I will be used. In this way, the $\lambda^{2}$-term controls the magnitude of the solution and prevents it from diverging. Thus (in what follows, the superscript ${}^{\left(LS\right)}$ will be omitted),

(4) $$\begin{array}{cc} J_{\lambda } & =J_{B}K^{T}{\left(KK^{T}+\lambda^{2}I\right)}^{-1}\\ \; & =J_{B}\mathrm{VS}{\left(S^{2}+\lambda^{2}I\right)}^{-1}U^{T} \end{array}$$

where

(5) $$K={\mathrm{USV}}^{T}$$

is the SVD decomposition of the blur matrix. Tikhonov regularization is a very efficient approach for 1D and 2D separable kernels. Non-separable 2D blurring kernels are less friendly to Tikhonov regularization. The blurring is given by

(6) $$J_{B}\left[i,j\right]=\sum_{k,m}J\left[i+m,j+k\right]K\left[m,k\right]$$

In principle, this equation can be rewritten in the form:

Ax=b

and thus it is amenable to Tikhonov regularization. However, the images should be then converted to 1D vectors such that a W×H image becomes a WH×1 vector. The blur matrix A becomes a WH×WH sparse matrix. It is clear that the storage and processing requirements are significantly higher than for a separable case (if W=H=256, we have $2^{16}$ values in x and $2^{32}$ values in A).

#### Wiener Regularization

A well-known alternative way to solve the blurring Equation (6) is to move to Fourier space, where the equation becomes simply

(7) $$J_{B}\left(k\right)=J\left(k\right)K\left(k\right)+\eta \left(k\right),$$

with η(k) being the Fourier transform of image noise. Wiener [26] proposed a solution to this problem in the context of stationary time series. His solution applied to the domain of image deblurring has the following form:

(8) $$J_{W}\left(k\right)=\left[\frac{K^{*}\left(k\right)}{{\left|K(k)\right|}^{2}+\alpha^{2}\left(k\right)}\right]J_{B}\left(k\right).$$

The expression in parentheses is called *the Wiener Filter* and the term α(k) is the inverse of the original signal SNR

(9) $$\alpha \left(k\right)=\left|\frac{\eta (k)}{J(k)}\right|.$$

Since the α-term is in general unknown, it is usually replaced by a constant, which is denoted by $\lambda^{2}$, so that

(10) $$J_{\lambda }\left(r\right)=\frac{1}{WH}\sum_{k}\left[\frac{K^{*}\left(k\right)}{{\left|K(k)\right|}^{2}+\lambda^{2}}\right]J_{B}\left(k\right)e^{ikr},$$

where $K^{*}\left(k\right)$ is the complex conjugate of K(k). Equation (10) will be henceforth referred to as the *Wiener-regularized solution*.

### 2.2. Optimal Regularization Parameter—MSE Approach

For real images, analytical estimation of the optimal value for λ is not possible. Thus, the following experiment is performed:

1. Take an image
2. Design a blur kernel; blur the image; add noise
3. Perform regularized deconvolution using different values of λ
4. For each value of λ, calculate the mean squared error (MSE) between the deblurred image and the original one

This procedure is repeated for a set of images with varying illumination, content, size and texture. First, a blur kernel is fixed and plot the mean error for various values of λ. Then, the analysis is performed for different blur kernels. In this section, one-dimensional kernels and Tikhonov-regularized deblurring are used. This approach can be readily extended to include 2D kernels and Wiener-regularized deblurring.

**Figure 1:**
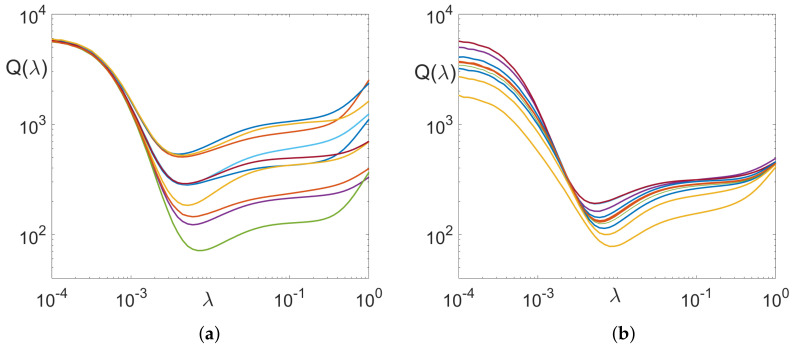
Deblurring Error for (**a**) single kernel, different images; (**b**) single image, various kernels.

For a given image J and a blur kernel K, the mean squared deblurring error *Q* is given by

(11) $$Q\left[K;\lambda ,\eta \right]=\frac{1}{\Omega }{∥\left(JK+\Xi \right)K_{\lambda }^{†}-J∥}^{2},$$

where Ω is the number of pixels in the image, Ξ is the random Gaussian noise matrix of the same dimensions as the image

(12) $$\Xi_{ij}∼N\left(0;\eta \right),$$

and

(13) $$K_{\lambda }^{†}=K^{T}{\left(K^{T}K+\lambda^{2}L^{T}L\right)}^{-1}$$

is the regularized deblur matrix. Figure 1a shows a typical dependence of *Q* on λ calculated for a set of images $\left\{J\right\}$. Gaussian blur with σ=30 is used, and the added noise intensity is η=0.1.

**Figure 2:**
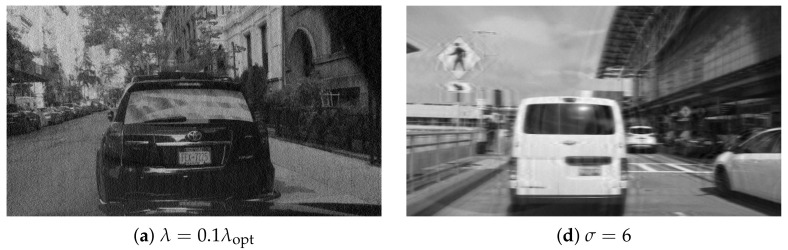

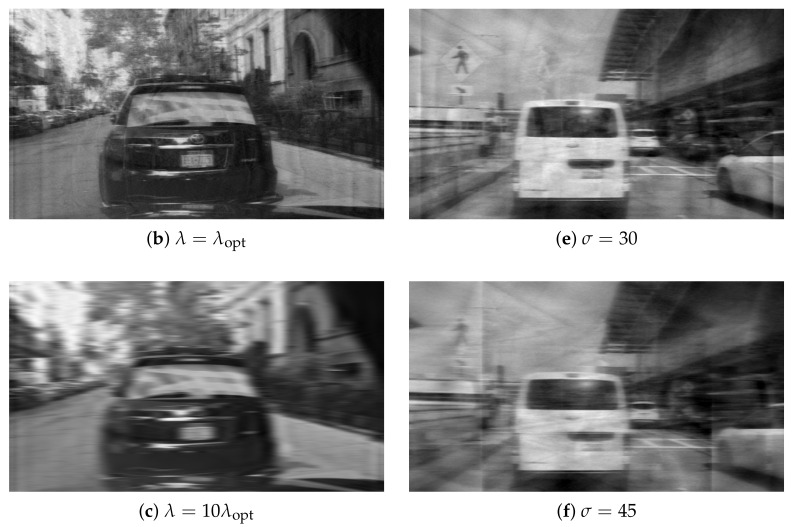
Deconvolved images: (**a**–**c**) single blur kernel, different λ values; (**d**–**f**) different blur kernels, $\lambda =\lambda_{\mathrm{opt}}$ for each kernel.

First, one can see that the error varies over several orders of magnitude, it is large for small and large λ, and reaches a minimum for some intermediate value. For λ smaller than the optimum value $\lambda_{\mathrm{opt}}$, the error comes mainly from noise amplification; for larger values, it comes from image residual blur (Figure 2). The actual numbers vary for different images, however, the main qualitative characteristics of the dependency Q(λ) remain the same. Now, the above procedure is repeated for different blur kernels. The same Gaussian blur as above is used, with σ uniformly distributed between 5 and 50. The results are shown in Figure 1b. As one can see, both the minimal attainable error and the optimal value $\lambda_{\mathrm{opt}}$ vary as the size of the blur is varied. The variation of $\lambda_{\mathrm{opt}}$ is not large (∼30%), thus, a single value of $\lambda_{\mathrm{opt}}$ can be used for most cases.

In practice, the reference images are not available, so Q(λ) cannot be minimized to estimate the optimal value for λ. Correct estimation of λ has been a rather active research topic for quite a few decades [27], and produced several semi-heuristic approaches. Two most popular methods: Generalized cross-validation (GCV) [28] and the L-Curve [29] usually produce values of λ that are within an order of magnitude of Q(λ)-minimizing value. The resulting deconvolution is therefore sub-optimal. In Section 3.2 it will be shown how the problem of λ estimation can be solved using machine learning techniques.

### 2.3. Common Artifacts in Image Deblurring

Regularized image deblurring techniques described above belong to a category of deconvolution algorithms appearing in various signal estimation contexts [25]. More often than not, such regularized solutions exhibit artifacts of varying severity. In addition to random noise and over-smoothing, the most common types of artifacts appearing in deconvolved images are ringing and boundary artifacts. Both types appear as quasi-periodic modulations of image intensity. Sometimes, both types of artifacts are referred to as “ringing”, although their origins are somewhat different.

*Ringing artifacts* are a manifestation of the well-known Gibbs phenomenon: overshoots of Fourier sums at signal discontinuities. Regularization effectively removes high frequencies from the reconstructed image, thus exacerbating the phenomenon. In practice, ringing is more visible around objects surrounded by non-textured background such as the sky or uniform highway surface.

In a blurred image, pixels close to the image boundary contain information from the area outside of the image borders. Thus, deconvolution will not have this information available for proper reconstruction. As a result, the effect of missing boundary pixels propagates throughout the image, sometimes corrupting it beyond recognition. This phenomenon is referred to as *boundary artifacts*. Typical modulation length will be of the order of PSF size, since this is the extent of unknown pixels to be filled in. At the same time, intensity modulation frequency associated with ringing depends on PSF spectrum rather than on its size. Deconvolved images in Figure 2 clearly exhibit boundary artifacts of varying intensity.

Many tricks were proposed in the past to properly handle deconvolution artifacts. For example, a standard approach to fight boundary artifacts is to design suitable boundary conditions—from naïve pixel replication to sophisticated variation-minimization tiling [30]. Other approaches [31,32,33,34] incorporate artifact prevention into reconstruction algorithms. Finally, some researchers have proposed to post-process deconvolved images for detection and removal of unwanted spatial modulations [35].

## 3. Deblurring with Deep Learning

This section describes how deep neural networks can be trained to remove deconvolution artifacts. Following [23], throughout the rest of this work a two-step approach to deblurring is adopted:

1. Perform regularized deconvolution of the blurred image
2. Pass the deconvolved image through a deep neural network to remove residual artifacts.

In order for this approach to work, one needs to select a good value for λ for the first step, and a suitable network architecture for the second step.

This section is organized as follows. First, the two-step approach is described for the case of fixed input noise. It is then extended to incorporate joint parameter optimization for overall deblurring performance improvement. Next, it is demonstrated that there can be no single optimal parameter λ that could handle regularized deconvolution of variable-noise input. Finally, as a solution, the two-step approach is generalized to include two deep neural networks: one for estimating the regularization parameter λ from the input images, and another one to enhance the intermediate deconvolved images.

### 3.1. Known Input Noise

As shown in Section 2.2, for a fixed level of noise in the blurred set, slight variations in the value of λ around $\lambda_{\mathrm{opt}}$ do not increase the deblur error significantly. It is reasonable to assume that these variations can be handled by subsequent processing in the DNN. Therefore, the deblur error is calculated using (11) averaged over the training set:

(14) $$\langle Q\left[K;\lambda ,\eta \right]\rangle =\frac{1}{N_{T}\Omega }\sum_{J\in {\left\{J\right\}}_{T}}{∥\left(JK+\Xi \right)K_{\lambda }^{†}-J∥}^{2},$$

where ${\left\{J\right\}}_{T}$ is the training set and $N_{T}$ is its size. The parameter λ is set at the value that minimizes $\langle Q\left[K;\lambda ,\eta \right]\rangle$:

(15) $$\lambda =\lambda_{\mathrm{opt}}={\mathrm{argmin}}_{\lambda }\langle Q\left[K;\lambda ,\eta \right]\rangle$$

#### 3.1.1. Neural Network Architectures

Throughout the rest of this paper, the post-processing neural network will be referred to as the image enhancement network (IEN).. Unlike the well-known classification-oriented architectures, input and output of the IEN should have identical dimensions. Two simple choices for building such a network would be either to keep the image dimensions uniform in all the layers, or to use an encoder-decoder architecture [36]. In this section, both options are examined. Also, in both cases, the aim is to estimate the residual between a pre-deconvolved image and the corresponding sharp image, similarly to [24].

##### Uniform Width CNN

The first tested IEN architecture is the uniform width (UW) CNN. The input image dimensions are halved in the first (7×7) convolutional layer producing $N_{F}$ feature images. The first layer is followed by $N_{L}$ (3×3) convolutional layers with the same number of features $N_{F}$, and then a (7×7) convolution transpose layer restoring the image dimensions to their original value. The output of the convolution transpose layer is added to the input image so that the network learns to extract the residual image from the input. Every convolution layer is followed by batch normalization and a ReLU activation function.

##### U-Net

The second examined IEN architecture is a variant of the U-Net [37] proposed by Jin et al. [16] for solving various kinds of ill-posed inverse problems in imaging. In particular, it was used for removing reconstruction artifacts from medical imagery. Network hyper-parameters are chosen such that input image dimensions should be divisible by $2^{6}$.

#### 3.1.2. Joint Parameter Optimization

It was previously shown that CNNs do a decent job removing artifacts generated by regularized deconvolution for a carefully chosen value of λ. This value strikes the balance between producing a deconvolved image that is too noisy and one that is too blurry. However, it is known that DNNs have a capacity for residual denoising and deblurring of images [38]. Therefore, the following question can be asked: Is it possible to move away from $\lambda_{\mathrm{opt}}$ and still improve the overall performance of the system? In order to do that, the regularized deconvolution (RD) block should be treated as an optimizable module. Then, λ would be initialized at the value found in (15), and would vary together with the IEN weights during the end-to-end performance optimization process.

To proceed with the joint numerical optimization, it must be specified how to calculate the derivatives of the neural network cost with respect to λ. Within a standard gradient-based cost function optimization scheme such as stochastic gradient descent (SGD) optimization, the update relation for any NN parameter θ (from step n−1 to step *n*) is:

(16) $$\theta_{n}=\theta_{n-1}-\epsilon \left(\frac{\partial L}{\partial \theta }\right),$$

where L is the cost function of the NN and ϵ is the learning rate. Typically, since the dependence of the cost function on any NN parameter θ is manifested through a chain-like dependence scheme, the required gradients are calculated by a back-propagation algorithm. Specifically, if

(17) $$L=g_{0}\left(g_{1}\left(g_{2}\left(\ldots \left(g_{N}\left(\theta \right)\right)\right)\right)\right)$$

Then

(18) $$\frac{\partial L}{\partial \theta }=\frac{\partial L}{\partial g_{N}}\frac{\partial g_{N}}{\partial \theta }=\frac{\partial L}{\partial g_{N-1}}\frac{\partial g_{N-1}}{\partial g_{N}}\frac{\partial g_{N}}{\partial \theta },$$

and so on. The overall cost as a function of the regularization parameter λ is

(19) $$L=L(J_{D}\left(\lambda \right))$$

with J(λ) being the input of the CNN, i.e., the regularized deblurred image.

Now, since for the Tikhonov-regularized deconvolution case

(20) $$J_{D}\left(\lambda \right)=J_{B}K^{†}\left(\lambda \right)=J_{B}VS{\left(S^{2}+\lambda^{2}I\right)}^{-1}U^{T}$$

it follows that

(21) $$\frac{\partial L}{\partial \lambda }=-2\lambda \left(\frac{\partial L}{\partial J_{D}}\right)J_{B}VS{\left(S^{2}+\lambda^{2}I\right)}^{-2}U^{T},$$

so that the update scheme for λ will be

(22) $$\Delta \lambda_{n}=2\epsilon \lambda_{n-1}\left(\frac{\partial L}{\partial J_{D}}\right)J_{B}VS{\left(S^{2}+\lambda_{n-1}^{2}I\right)}^{-2}U^{T}.$$

The input gradients $\partial L/\partial J_{D}$ are fed back from the CNN part of the overall NN scheme.

The above idea can be readily extended to the Wiener filter case. Since

(23) $$J_{D}=\mathrm{IFFT}\left[J_{B}\left(k\right)\frac{K^{*}\left(k\right)}{{\left|K\left(k\right)\right|}^{2}+\lambda^{2}}\right],$$

it follows that

(24) $$\Delta \lambda_{n}=2\epsilon \lambda_{n-1}\left(\frac{\partial L}{\partial J_{D}}\right)\mathrm{IFFT}\left[\frac{J_{B}\left(k\right)K^{*}\left(k\right)}{{\left({\left|K\left(k\right)\right|}^{2}+\lambda_{n-1}^{2}\right)}^{2}}\right]$$

### 3.2. Noise-Adaptive Deblurring

#### 3.2.1. Deblurring Error for Varying Noise

In real life, noise levels of input images can vary quite significantly. A number of researchers tried to address this point previously. For instance, Schmidt et al. [39] proposed a MAP approach to non-blind deconvolution that incorporates noise estimation in the algorithm.

**Figure 3:**
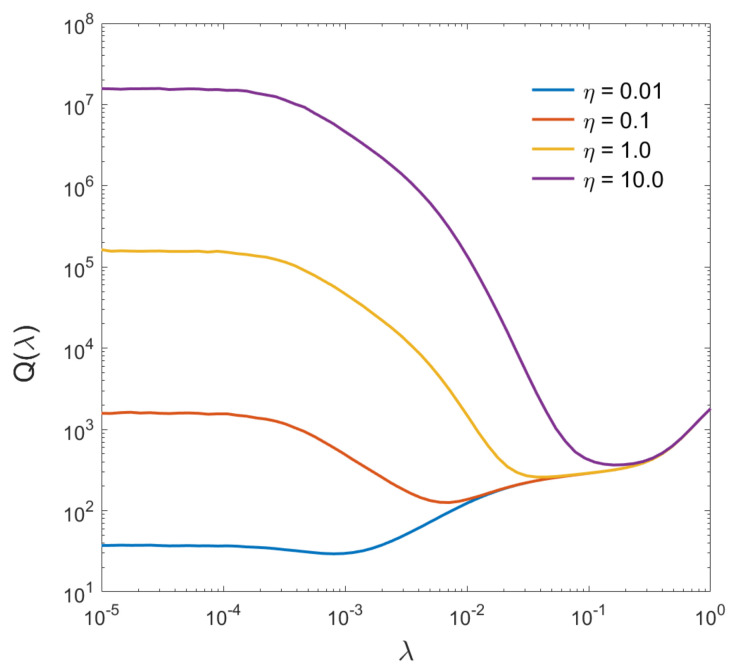
Deblur error for different levels of input noise.

To quantify the effect of noise variation on regularized image deconvolution, Figure 3 depicts the deblur error (11) as a function of λ for a random image from the training set, for noise levels varying over three orders of magnitude: from $\eta =10^{-2}$ to η=10.0. As before (Figure 1), the deblur error spans several orders of magnitude. The variation, however, is significantly larger for higher levels of noise, mainly to the left of $\lambda_{\mathrm{opt}}\left(\eta \right)$ due to practically unbounded noise amplification. On the other hand, the residual blur does not exhibit strong dependence on the noise, therefore, the graphs tend to converge for larger values of λ. The minima of $Q_{\eta }\left(\lambda \right)$ appear rather flat in the context of overall error variation, however, they are sufficiently pronounced, more so for higher levels of noise. This is to be expected, since for low noise levels, regularized deconvolution is quite tolerant to the value of λ.

The main observation that is rather obvious from Figure 3, is that there can be no single optimal parameter $\lambda_{\mathrm{opt}}^{*}$ that can handle regularized deconvolution of variable-noise input. The deconvolved images will be either buried in noise (if $\lambda_{\mathrm{opt}}^{*}<\lambda_{\mathrm{opt}}\left(\eta \right)$) or will have excessive residual blur (if $\lambda_{\mathrm{opt}}^{*}>\lambda_{\mathrm{opt}}\left(\eta \right)$). It can be predicted with a high degree of confidence, that repeating training procedures from the previous section for variable-noise input will strongly bias the value of $\lambda_{\mathrm{opt}}^{*}$ towards higher values therefore producing over-smoothed images. On the other hand, it is also clear that the minimal attainable error $Q[\lambda_{\mathrm{opt}}\left(\eta \right)]$ does not vary as much for different levels of noise. Thus, even for strong noise, if a corresponding $\lambda_{\mathrm{opt}}$ can be found, reasonable output may still be produced.

**Figure 4:**
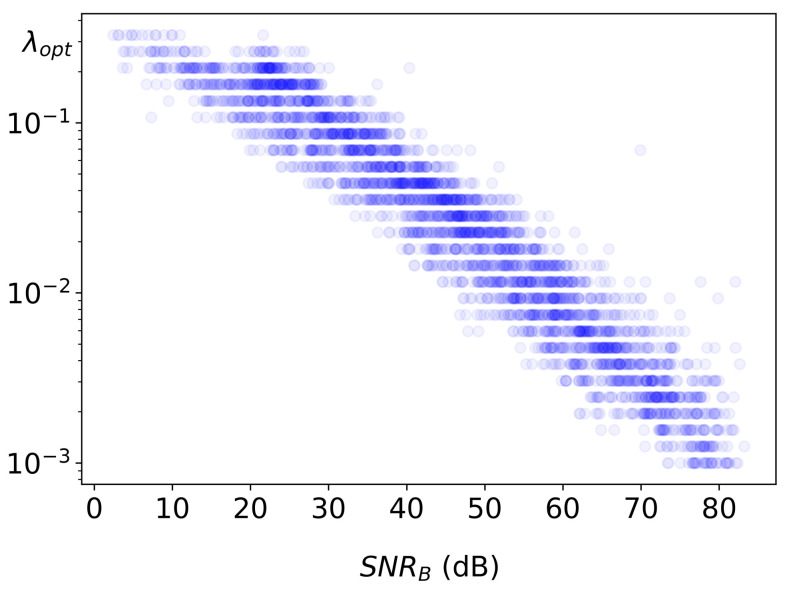
Dependence of $\lambda_{\mathrm{opt}}$ on the input $SNR_{B}$.

Next, the connection between the input noise and the corresponding $\lambda_{\mathrm{opt}}$ calculated from Equation (15) is examined. To quantify the amount of noise in the corrupted image a metric called $SNR_{B}$ is used. It is defined as a ratio between average blurred image intensity and added noise intensity. Note that $SNR_{B}$ is by no means an image quality metric; it is merely a normalized characteristic of noise contamination for input images. Figure 4 shows a log-log plot of $SNR_{B}$ vs $\lambda_{\mathrm{opt}}$ for 100 blurred images corrupted with 50 different noise levels each, with intensities ranging between $\eta =10^{-3}$ and η=10.0. Estimated correlation for $SNR_{B}$ and $\lambda_{\mathrm{opt}}$ data is found to be very strong (–0.98), and the linear fit to the log-log data suggests the following empirical dependence:

(25) $$\lambda_{\mathrm{opt}}\propto {\left(SNR_{B}\right)}^{-3/4}$$

However, for this relation to be useful, a reliable estimate for $SNR_{B}$ is needed. Unfortunately, SNR estimation is in itself not a trivial problem. There are a number of ways to estimate SNR of an image without a reference [40,41], but using them will only increase the uncertainty of $\lambda_{\mathrm{opt}}$ estimation.

#### 3.2.2. The General Idea

If one could find a way to generate a proper value of λ for any blurred and noisy image, then, in principle, the problem will be reduced to the two-step solution described above.

**Figure 5:**
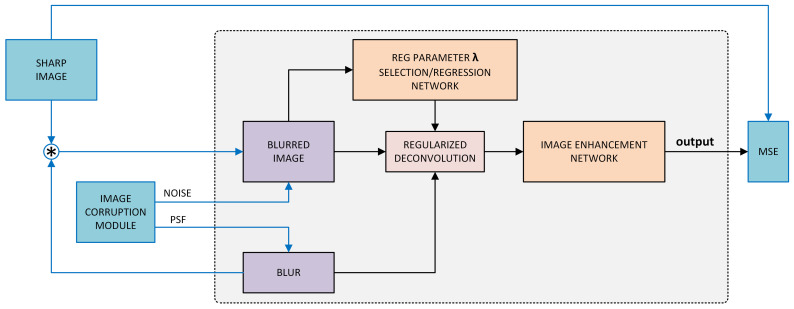
The proposed concept for noise-sensitive solution. Blue blocks/arrows describe the method for self-supervised end-to-end training of the system.

It is therefore proposed to train a deep neural net to estimate $\lambda_{\mathrm{opt}}$ from the blurred image itself. Thus, the overall deblurring system will include two DNNs - the first network for estimating $\lambda_{\mathrm{opt}}$, and the second one for image enhancement after regularized deconvolution, as described in Section 3.1. The first network will be henceforth referred to as regularization parameter estimation network, or *RegParamNet*. Figure 5 shows the concept of the proposed solution. A blurred image is fed into a network that generates a value for λ. Then, using this value and the known PSF, the image is deconvolved and the result is fed into the IEN.

#### 3.2.3. RegParam Network Architecture

**Figure 6:**
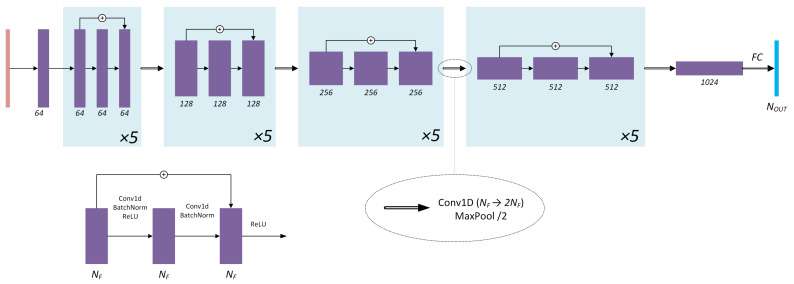
RegParamNet architecture.

As Hansen points out in [9], blurred images have much faster decaying singular value (SV) spectra than corresponding sharp ones. Adding noise boosts the small SV part of the spectrum so that noisy blurred images decay have SV spectrum decaying slower than clean ones. Following Hansen, it is assumed that the SV spectrum of an image contains enough information regarding the relationship between high-frequency noise and high-frequency features in the image. Thus, the aim is to build a DNN that would take SV spectrum of an image as an input and would output $\lambda_{\mathrm{opt}}$. The ResNet architecture [42] is taken as a reference, and its 1D analogue is built: while ResNet works with 2D images and 2D convolutions, RegParamNet processes 1D inputs. Figure 6 shows the final architecture that was chosen after several rounds of experimenting with network hyperparameters, such as depth and number of features. The input signal is a 1D vector composed of image SV logarithms. The input is then converted in the first convolutional layer to 64 feature vectors. The next four stages of RegParamNet are cascades of five residual blocks as shown in Figure 6. There is a feature number doubling convolutional layer and a max-pooling layer following each cascade. Finally, 1024 feature vectors are fed into a fully connected layer to produce the output vector.

Here, two options for $\lambda_{\mathrm{opt}}$ estimation using RegParamNet are considered:

1. Direct regression of $\lambda_{\mathrm{opt}}$
2. Selection of $\lambda_{\mathrm{opt}}$ from a set of values.

In both cases, network training consists of two steps. First, RegParamNet is pre-trained to generate $\lambda_{\mathrm{opt}}$ that minimizes the deblur error $Q_{\eta }\left(\lambda \right)$ of (11). Then, it is connected to the IEN as shown in Figure 5 for end-to-end (E2E) system training.

#### 3.2.4. RegParamNet Training Schemes

##### Training Data Generation

**Figure 7:**
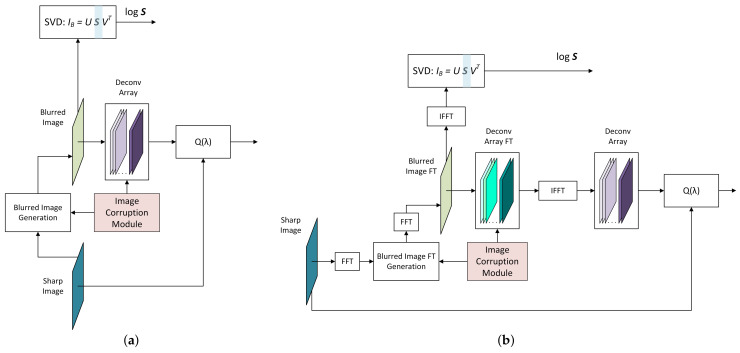
RegParamNet training data generation: (**a**) Tikhonov deconvolution; (**b**) Wiener deconvolution.

RegParamNet should be able to handle a wide range of noise magnitudes and blur kernels. For this purpose, the image corruption module (ICM) is introduced, generating blur and noise in a random manner for each training iteration. For instance, 1D blur kernels (Figure 7a) are generated according to the following scheme:

(26) $$\begin{array}{cc} K_{n} & =\frac{p_{n}}{\sum_{m}p_{m}},\;p_{n}=e^{-{\left(n-\mu \right)}^{2}/2\sigma^{2}},\;0\leq n\leq L-1\\ L & ∼U\left(16,64\right)\;\sigma ∼U\left(\frac{L}{4},L\right)\;\mu ∼U\left(\frac{L}{4},\frac{3L}{4}\right) \end{array}$$

In addition, ICM randomly picks a value η from a log-uniform distribution such that $10^{-2}\leq \eta \leq 10.0$

(27) $$\log_{10}\eta ∼U\left(-2,1\right),$$

and adds random noise of this magnitude to the blurred image. For 2D Wiener deconvolution, blur functions are generated as 2D Gaussians with random covariance:

(28) $$K_{n}=\frac{p_{n}}{\sum_{n}^{\prime }p_{n}^{\prime }},\;p_{n}=e^{-\frac{1}{2}n^{T}\Gamma^{-1}n},$$

where

(29) $$-\frac{L_{x,y}}{2}\leq n_{x,y}<\frac{L_{x,y}}{2}.$$

and

(30) $$\begin{array}{c} \Gamma_{xx}=\alpha L_{x}L_{y},\;\Gamma_{yy}=\beta L_{x}L_{y}\\ \Gamma_{xy}=\Gamma_{yx}=0.9\delta \sqrt{\Gamma_{xx}\Gamma_{yy}}\\ \alpha ,\beta ∼U(0,1),\;\delta ∼U(-1,1) \end{array}$$

The factor 0.9 in $\Gamma_{xy}$ ensures the invertibility of Γ; $L_{x}=64$ and $L_{y}=32$ were used.

For each training iteration, ICM provides data to generate blurred image from a sharp one. Using blur data from ICM and a vector of λ-values, an array of deconvolved images (DeconvArray) is created. The entries of λ-vector are incremented exponentially such that

(31) $$\log_{10}\lambda_{i}=-3+\frac{3}{N_{\lambda }-1}i,\;0\leq i\leq N_{\lambda }-1$$

Calculating MSE of DeconvArray relatively to the sharp image gives us the function Q(λ). At the same time, SVD of the blurred image is calculated and the array of SV logarithms is fed to the RegParamNet. The schemes for Tikhonov and Wiener cases are shown in Figure 7.

##### RegParamNet Modes

**Figure 8:**
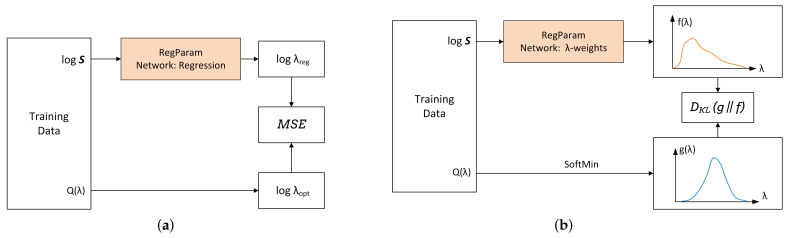
RegParamNet training: (**a**) Direct λ regression; (**b**) g(λ) approximation (“λ-weights”).

The scheme used for pre-training RegParamNet to directly regress $\lambda_{\mathrm{opt}}$ is shown in Figure 8a. The fully connected layer of RegParamNet outputs a single float number that is passed to MSE loss module together with $\log\lambda_{\mathrm{opt}}$. Regression of logλ is much more stable than regression of λ since for all practical purposes λ<1 and often it is very small.

An additional way to estimate $\lambda_{\mathrm{opt}}$ is to train the network to generate the function Q(λ) given the SV spectrum of the blurred image and find its minimum. In this case, the FC layer of RegParamNet produces a real-valued vector, the size of which equals the length of the λ-vector. Since Q(λ) can reach extremely large values, it is more practical to work with another function g(λ):

(32) $$g\left(\lambda \right)=\mathrm{softmin}\left[Q(\lambda )\right]=\frac{\exp\left[-Q(\lambda )\right]}{\sum_{\lambda^{\prime }}\exp\left[-Q(\lambda^{\prime })\right]}$$

Then,

(33) $$\lambda_{\mathrm{opt}}=\mathrm{argmin}\left[Q(\lambda )\right]=\mathrm{argmax}\left[g(\lambda )\right]$$

To train RegParamNet to generate a function similar to g(λ) a setup shown in Figure 8b is used. Blurred images and corresponding MSE vector Q(λ) are created like in the previous section. Then, Q(λ) is converted to g(λ) using (32). The output of RegParamNet passes through a softmax layer to produce a vector f(λ). Since both g(λ) and f(λ) vectors are normalized, they can be treated as probability distributions. A well-known similarity measure for two distributions is their Kullback–Leibler divergence [43]:

(34) $$D_{KL}\left[g∥f\right]=\sum_{\lambda }g\left(\lambda \right)\log\left[\frac{g(\lambda )}{f(\lambda )}\right]$$

$D_{KL}\left[g∥f\right]$ is always non-negative and is only zero when the two distributions are identical. Thus, setting $D_{KL}\left[g∥f\right]$ as the RegParamNet loss and minimizing it, the network is trained to approximate g(λ). Then, to use RegParamNet to estimate $\lambda_{\mathrm{opt}}$, one just needs to find the maximum of f(λ).

## 4. Experiments

### 4.1. Known Input Noise

#### 4.1.1. Training

For training, images from Berkeley DeepDrive (BDD) database [44] were used. Original images were converted to grayscale and down-scaled by a factor of 0.5 to produce 360×640 images. To make the images compliant with the U-Net architecture described above, they were further cropped to 320×640. Sharp images were blurred using a variety of 1D Gaussian blur kernels with σ varying from 30 to 64. Blurred images were then additionally corrupted by Gaussian noise with RMS intensity η=0.1. The optimal regularization factor for η=0.1 was found by the technique described in Section 2.2 to be $\lambda_{\mathrm{opt}}=7.5\times 10^{-3}$. The blurred images were deconvolved using Tikhonov regularization. The deconvolved images were used as inputs to the CNNs. MSE between CNN output and the original sharp images was used as the network loss.

The networks were implemented in the PyTorch framework [45] and initialized using Kaiming-normal initialization [46]. The networks were trained on 40,000 images and validated on 10,000 images for 30 epochs. SGD algorithm was used, with initial learning rate of $10^{-3}$ that was reduced to $10^{-4}$ after 15 epochs.

#### 4.1.2. Quality Metrics

Assessment of image quality is not a very well defined task, especially in the absence of reference images  [47]. Depending on the final goal of image processing chain in question, different metrics may be more or less well suited for quantifying algorithm performance. Tasks like image compression, color balancing, or de-noising can be (and usually are) evaluated differently by a group of human testers compared to some objective metric. In our rather reduced scope, deblurring is treated as a standalone module; also, reference images are available for testing. Therefore, deblurred image quality is assessed using the commonly accepted peak signal-to-noise ratio (PSNR) metric. For the sake of completeness, structural similarity (SSIM)  [48] for deblurred images was evaluated as well; SSIM values correlate rather well with PSNR values and strongly support the main findings.

#### 4.1.3. Results

For testing, a separate subset of 10,000 images from the BDD dataset was used. The images were converted to grayscale, resized and cropped as described in Section 4.1.1.

Three CNN configurations were tested: uniform width (UW) architecture with $N_{F}=64$ and $N_{F}=128$ and residual U-Net architecture. Mean PSNR values are summarized in Table 1.

**Figure 9:**
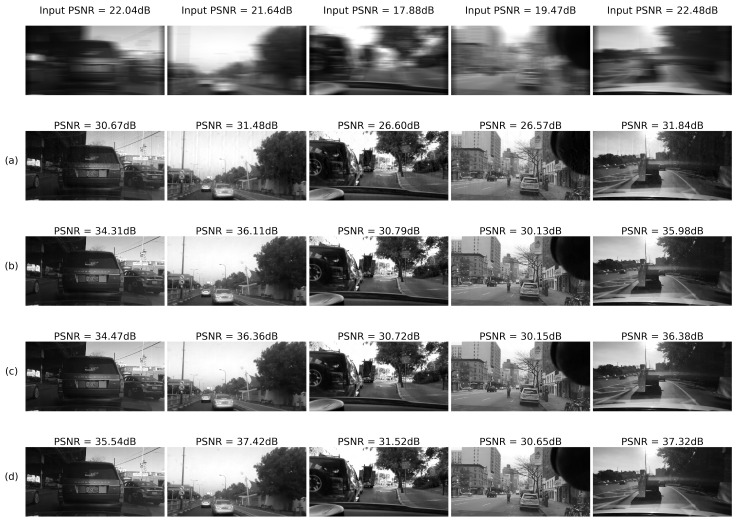
Two-step image deblurring for a set of images: (**a**) Tikhonov Deconvolution with $\lambda =7.5\times 10^{-3}$; (**b**) Initial + UW-64; (**c**) Initial + UW-128; (**d**) Initial + residual U-Net.

First of all, it is clear that just using a deep neural net increases the image PSNR by about 4 dB on average. Figure 9 shows that this large PSNR increase can be mainly attributed to deconvolution artifact removal and some image denoising. Secondly, the difference between UW-64 and UW-128 is not significant. It is also clear that residual U-Net outperforms the UW networks. Thus, throughout the rest of this work, the residual U-Net architecture is used.

#### 4.1.4. Joint Parameter Optimization

The regularized deconvolution (RD) module was implemented using PyTorch framework using AutoGrad functionality [49]. The regularization parameter λ was initialized at $\lambda_{\mathrm{opt}}=7.5\times 10^{-3}$. The entire network (RD+residual U-Net) was trained using the procedure outlined in Section 4.1.1.

The statistics for 10,000 test images are outlined in Table 2 below. The new value of the regularization parameter is $\lambda_{\mathrm{opt}}^{*}=3.3\times 10^{-3}$, so that we expect the deconvolved image to be on the noisy side. Indeed, the mean PSNR for deconvolved images drops by 2.5 dB relatively to $\lambda_{\mathrm{opt}}=7.5\times 10^{-3}$ case. However, the IEN is able to remove the additional noise rather effectively demonstrating better overall performance than for $\lambda =\lambda_{\mathrm{opt}}$.

**Figure 10:**
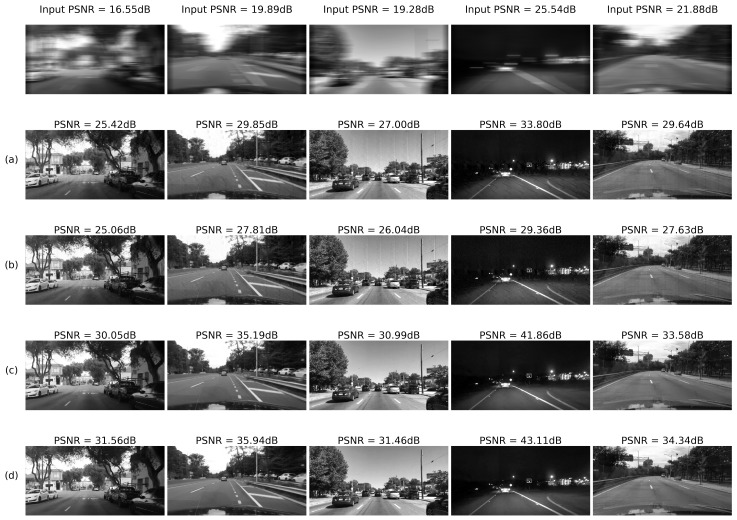
Effect of joint training for a set of images: (**a**) Deconvolved image with $\lambda =\lambda_{\mathrm{opt}}$; (**b**) Deconvolved image after joint training; (**c**) Two-step deblurring (**d**) Two-step deblurring after joint training.

Finally, Figure 10 demonstrates image quality improvement from deconvolved images, through standalone two-stage image enhancement to the joint end-to-end system optimization.

### 4.2. Noise-Adaptive Deblurring

#### 4.2.1. RegParamNet Training

Input images from the BDD database were converted and down-scaled as described in Section 4.1.1. The networks were trained on 40,000 images and validated on 10,000 images for 30 epochs. ADAM optimizer algorithm was used with initial learning rate of $10^{-4}$ that was adaptively reduced by a ReduceLROnPlateau scheduler. For each mini-batch, a blur kernel was generated as described in Training Data Generation Section and a value of η is randomly picked such that $\log_{10}\eta ∼U\left(-2,1\right))$.

##### Direct λ Regression

**Figure 11:**
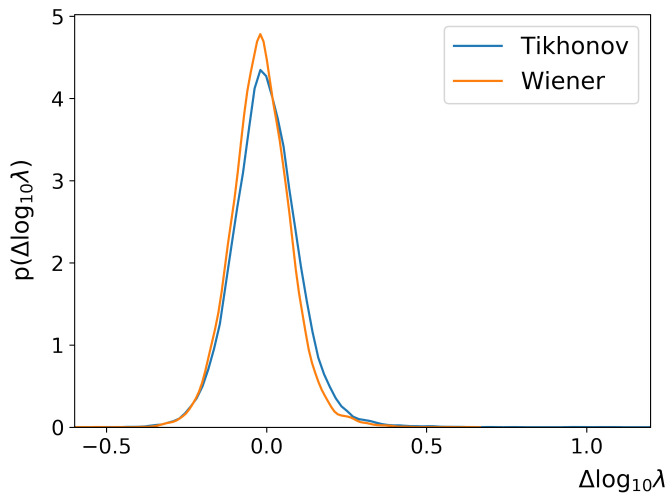
Direct $\lambda_{\mathrm{opt}}$ regression results.

Figure 11 shows the error distribution function of regularization parameter logarithm for the direct regression mode. One can see that RegParamNet is able to directly infer $\log_{10}\lambda_{\mathrm{opt}}$ for both Wiener and Tikhonov regularization cases, with overall standard deviations for $\log_{10}\lambda_{\mathrm{opt}}$ of ∼0.1. This corresponds to $\lambda_{\mathrm{opt}}$ accuracy of 20–25%; an error of this magnitude has a relatively insignificant effect on the deconvolved image quality.

##### λ-Weight Array Generation

**Figure 12:**
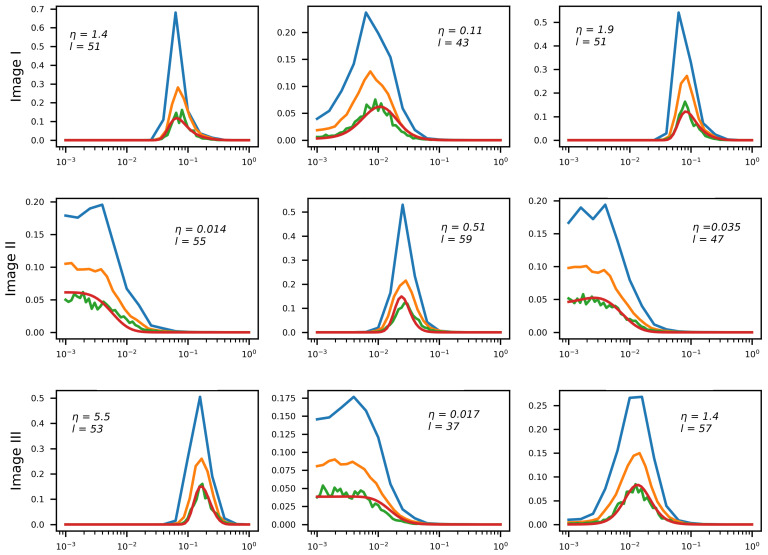
λ-weight array generation for various levels of input blur and noise (1D blur). Horizontal axis: λ values. Red Line: reference for 64 array entries. Blue/orange/green lines—generated arrays for 16/32/64 array entries, respectively.

Figure 12 shows the results of λ-weight array generation for several test images and several values of noise magnitude η from the range $10^{-2}\leq \eta \leq 10.0$. The networks generate λ-weight arrays for $\;N_{\lambda }=16,\;32,\;64$. The resulting arrays approximate the target function softmin[Q(λ)] with a similar degree of precision (height difference stems from f(λ) normalization; the peak approximately halves when $N_{\lambda }$ doubles).

#### 4.2.2. End-to-End System Training

**Figure 13:**
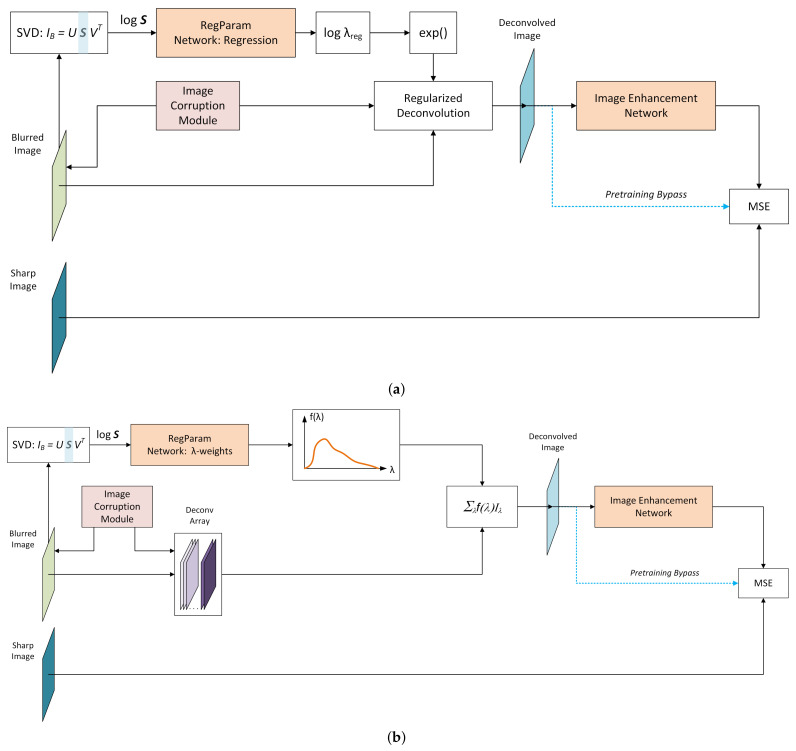
E2E system training schemes: (**a**) Regression RegParamNet; (**b**) λ-weight RegParamNet.

After the pre-training is finished, output of RegParamNet is connected through a regularized deconvolution (RD) module to the input of the image enhancement network. Specifically, a regressed value of $\lambda_{\mathrm{opt}}$ or a value of λ that maximizes f(λ) could be taken, used for regularized deconvolution and then the output can be fed into the IEN. However, as was shown in the previous section, $\lambda_{\mathrm{opt}}$ that minimizes Q(λ) does not necessarily lead to the best system performance. Thus, it is desirable to connect RegParamNet to IEN and train the two networks jointly. Figure 13 describes the overall training setup for both $\lambda_{\mathrm{opt}}$ regression and λ-weight cases.

Connecting λ-weight RegParamNet to the RD module and, eventually, to the IEN, for the purpose of joint training is less straightforward than in the regression case. The reason for this is that ArgMax is not an analytical function and thus cannot be easily integrated into the back-prop optimization framework. Thus, the following estimator $\hat{I}$ for the deconvolved image is proposed:

(35) $$\hat{I}=\sum_{\lambda }f\left(\lambda \right)I_{\lambda }$$

where $I_{\lambda }$ is the DeconvArray entry corresponding to parameter λ. Then, joint end-to-end (E2E) training as shown in Figure 13b can reshape f(λ) and change the weights of IEN so that the overall system performance improves.

From the practical point of view, a three-step training approach was found to produce best results. First, for each setup in Figure 13, a pre-trained RegParamNet was connected to the RD and the IEN was bypassed. After this step, f(λ) became more peaked and narrow around the optimal value. Then, RegParamNet was frozen and the IEN was pre-trained on RegParamNet outputs. Finally, RegParamNet was unfrozen to allow full E2E training. E2E training was implemented using λ-weight approach for both 1D and 2D deblurring; direct $\lambda_{\mathrm{opt}}$ regression was implemented for 1D deblurring only; we are currently working to extend it to the 2D (Wiener) case as well. For each step, the training was performed as described in Section 4.2.1.

#### 4.2.3. Results: Statistics

The system was tested on 10,000 images from the BDD database for randomly generated blur and noise magnitudes as described above. Processed image quality was tested in the following configurations:

(a) RD output before E2E training
(b) RD output after E2E training
(c) IEN output before E2E training
(d) IEN output after E2E training

**Figure 14:**
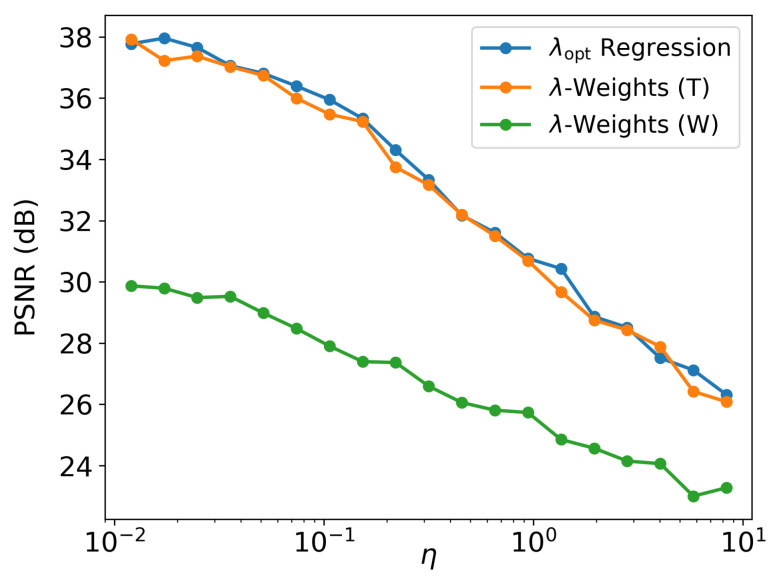
Mean result PSNR as a function of input noise.

PSNR and SSIM were used as the quality assessment metrics. Table 3 summarizes the corresponding mean PSNR/SSIM values. One can see that, for the 1D case, λ-weight and direct regression RegParamNets perform quite well across all levels of input noise and blur. Thus, from the practical point of view, direct $\lambda_{\mathrm{opt}}$ regression could be preferable since it is more lightweight. In addition, since 2D blur is in general much stronger than 1D blur, final PSNR for Wiener deblurring cases is substantially lower than for 1D case. Figure 14 shows IEN E2E result PSNR as a function of input noise.

**Figure 15:**
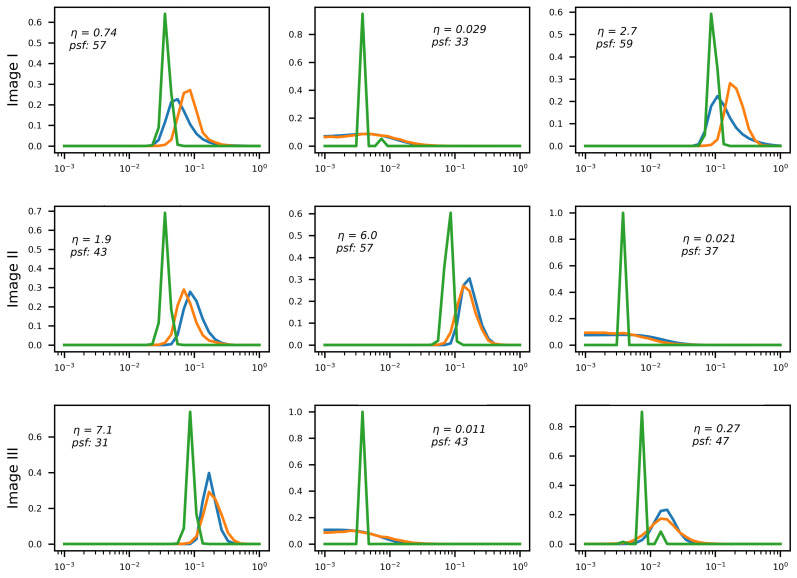
Effect of E2E training on λ-weight array generation; Blue line: reference g(λ), Orange: standalone f(λ), Green: f(λ) after E2E training.

It is noteworthy that the effect described in the previous section is clearly observable here as well: E2E training moves the RegParamNet away from the point where Q(λ) is minimized thus making the deconvolution sub-optimal. However, the IEN learns to compensate for this performance drop. In the 1D λ-weight case, it even improves the overall system performance by 1.3dB on the average. In other RegParamNet configurations, E2E training does not exhibit clear overall performance gain, though the IEN capacity is improved in all cases. Figure 15 clearly shows the effect of E2E training on the λ-weight array in the Tikhonov deconvolution case. The preferred λ values are typically smaller than in the standalone RegParamNet training, thus moving the pre-deconvolved images to the more noisy side of the standalone optimum. The only cases where this effect is not so clear are the ones with a very low level of input noise ($\eta ∼10^{-2}$), where the deconvolution is much less sensitive to the exact value of $\lambda_{\mathrm{opt}}$. One can therefore conclude that the IEN architecture in use has a better capacity for removing residual noise than for residual deblurring.

#### 4.2.4. Results: Images

**Figure 16:**
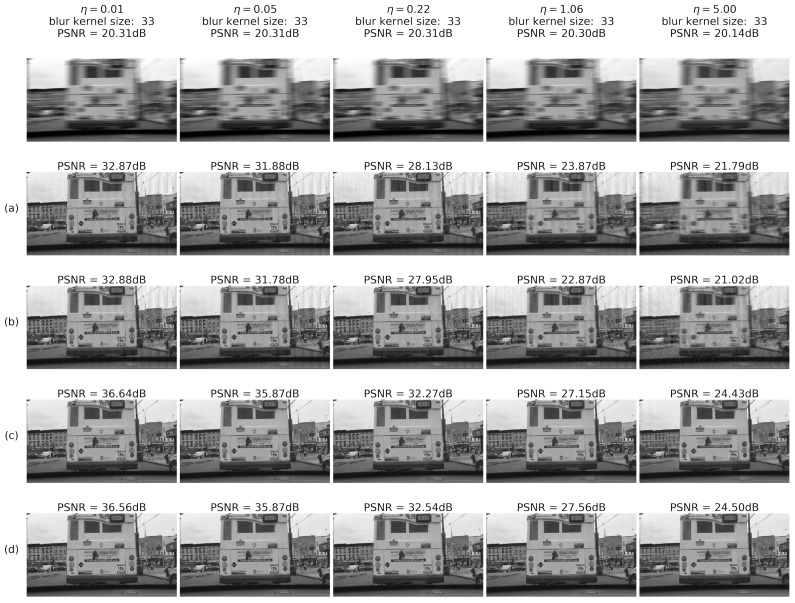
Deblurring using regression RegParamNet (Tikhonov): (**a**) RD output before E2E training; (**b**) RD output after E2E training; (**c**) IEN output before E2E training; (**d**) IEN output after E2E training.

**Figure 17:**
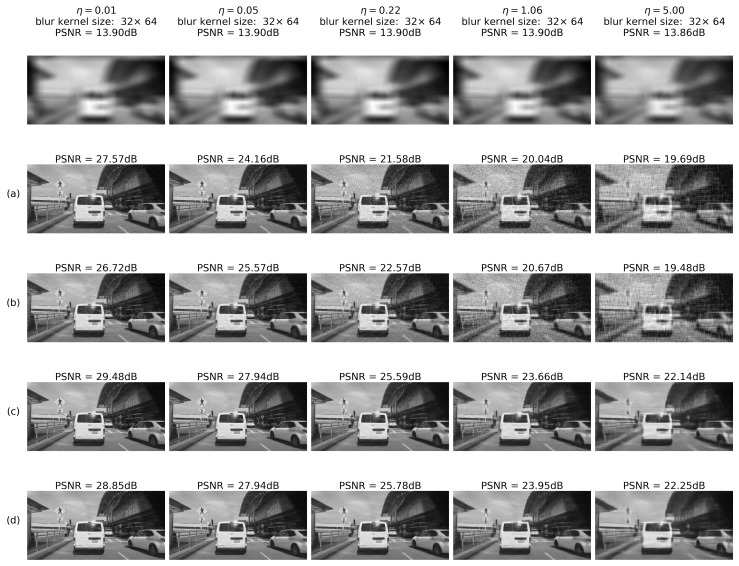
Deblurring using Wiener deconvolution: (**a**) RD output before E2E training; (**b**) RD output after E2E training; (**c**) IEN output before E2E training; (**d**) IEN output after E2E training.

To summarize, examples of noise-adaptive image deblurring for test images (best viewed on a soft copy. Additional high-resolution images are provided in the Supplementary Materials). are presented in Figure 16 and Figure 17. First, it can be seen that the system is capable of handling both strong input noise and large blur kernels. The deconvolution step is adequately regularized across the entire range of input noise intensities, and the subsequent image enhancement step removes most artifacts. To control noise amplification, regularization is naturally stronger for high input noise, resulting in less sharp output images. However, additional quality gain of 0.3–0.8 dB coming from E2E training is consistently observed on the high-noise side (rows (d) vs. (c) in Figure 16 and Figure 17). As described above, this gain is accompanied by a quality drop at the deconvolution step (rows (b) vs. (a) in Figure 16 and Figure 17). Second, one can also see that small features are clearly discernible even for η≃1.0. Stronger noise leads to eventual elimination of at least some of these features; however, larger objects are quite well defined and likely allow automatic classification.

#### 4.2.5. Results: Comparison to Other Approaches

In this section, the performance of the proposed method is compared to several previous approaches. The analysis follows the paper by Wang and Tao [24], and corresponding results from two additional leading works [50,51] are cited for reference as they appear there. The system was tested on Berkeley Segmentation Dataset [52] (BSD100). The following test scenarios were used:

- Gaussian kernel with spatial standard deviation equal to 1.6 and noise η=0.008 (denoted as GaussianA; η=0.01 in the proposed method)
- Gaussian kernel with spatial standard deviation equal to 3 and noise η=0.04 (denoted as GaussianB; η=0.05 in the proposed method)
- Gaussian kernel with spatial standard deviation equal to 5 and noise η=0.04 (denoted as GaussianC; η=0.05 in the proposed method)
- Square kernel with a side size of 19 and noise of η=0.01 (denoted as SquareA)
- Square kernel with a side size of 13 and noise of η=0.04 (denoted as SquareB; η=0.05 in the proposed method)

In addition, all 32 motion blur kernels from [53] were tested. The kernels were applied to test images from BSD100, followed by addition of noise. Two noise levels were tested: η=0.01 (MotionA), and η=0.22 (MotionB, compared to η=0.06 in [24]) was added. In each case average PSNR/SSIM was calculated over 3200 deblurred images.

Finally, the proposed method was tested at higher levels of input noise for the following Gaussian/Square configurations:

- Gaussian kernel with spatial standard deviation equal to 3 and noise η=1.06 (denoted as GaussianD)
- Gaussian kernel with spatial standard deviation equal to 5 and noise η=0.22 (denoted as GaussianE)
- Square kernel with a side size of 19 and noise of η=0.22 (denoted as SquareC)
- Square kernel with a side size of 13 and noise of η=1.06 (denoted as SquareD)

At these noise levels there are no data points from previous publications; for instance, Wang and Tao [24] reported that their experiments started breaking down at η∼ 0.1–0.2.

The results are summarized in Table 4. The current method is referred to as NANDB (Noise-Adaptive Non-Blind Deblurring). For the sake of completeness, the last column shows the performance of NANDB on 100 test images from the BDD set (denoted as NANDB*). It is rather clear that the proposed method outperforms other methods, often by as much as 3–5 dB in PSNR, especially for higher values of input noise. This can be attributed to the flexibility of the current method with respect to input noise intensity, whereas other works use “one-fits-all approach” in this regard. As was shown in Section 3.2, regularization parameter can be under- or overestimated by a factor of 1.5–2 without severe consequences for deconvolution. However, if input noise is allowed to vary over several orders of magnitude, fixed regularization parameters inevitably produce deconvolved images that are either over-smoothed or noisy beyond recovery. This also explains why other methods are applicable only in a narrow range of input noise intensities For instance, in [24] the system broke down already at η ∼ 0.1–0.2, as it was trained to handle noise in the range 0.008–0.06 only.

One can also see that the system performs even better if its usage is confined to automotive scenarios: The system trained on the BDD dataset exhibits significantly higher PSNR/SSIM values when tested on the BDD test images, as shown in the last column of Table 4. This improvement is likely due to a larger diversity of low- and high-level features present in the BSD100 dataset compared to BDD images. Thus, system performance is likely to improve if it is trained on large general datasets.

## 5. Summary

In this work, a systematic approach to non-blind deblurring is presented. Regularized deconvolution approaches are described as the means to control noise amplification, which is the main image degradation factor. Next, following previous work [23,24], removing deconvolution artifacts by a deep neural net (image enhancement network - IEN) is proposed. For the case of constant input noise, the two-step deblurring is extended by incorporating the regularized deconvolution module into a joint training framework. As a next step, the impact of input noise on the required amount of regularization is explored. To infer the regularization parameter (λ) values, RegParamNet, a novel 1D convolutional neural network is proposed. Two approaches for regularization parameter inference are explored (direct regression and λ-weights). Finally, a noise-adaptive non-blind image deblurring system is built by incorporating both RegParamNet and IEN into a common end-to-end training and inference framework.

The proposed system performs rather well on a wide range of large 1D/2D blur kernels, over three orders of magnitude of input noise. It is also found that end-to-end training biases the inferred regularization parameters downwards, so that deconvolved intermediate images are sharper, albeit noisier that for a standalone optimum. However, overall system performance is found to benefit from end-to-end training, especially for high levels of input noise (η = 0.2–10.0). Compared to other approaches, the system exhibits rather superior PSNR/SSIM performance even without domain adaptation, i.e., networks trained on automotive images (BDD) perform well on general type images (BSD100).

Although the present work suggests a principal solution to the problem of noise-adaptive non-blind deblurring, there are several research directions in which it can be extended. One rather important subject to be explored is the performance of perception algorithms on deblurred images. It has been noted before that some approaches to blind deblurring corrupt low-level features in processed images, thereby impacting detection and classification performance rather severely [3]. It is important to establish that no such effect takes place in our case. In addition, previous work suggests that imprecise knowledge of PSF produces effects akin to boundary artifacts [21]. Since PSF calibration or indirect estimation is always of limited precision, it is important to analyze the impact of PSF uncertainty on system performance, and to adjust the solution if needed. Also, it should be noted that current work presents a solution for gray-scale images only; an extension for color images should not be too difficult. Finally, certain pre- and post-processing stages can be added to the deblurring pipeline, such as preliminary de-noising of input images [54] or super-resolution [55] on output images. Both additions are likely to improve the overall system performance.

There is still much work to be done in order to convert the proposed deblurring scheme into a production-grade software system: architecture optimization, streamlining the processing flow, etc. For instance, it is clear that there is no need to infer regularization parameters for each captured frame, since the SNR changes at a much lower rate. However, automotive scenarios include abrupt scene illumination changes (e.g., when entering or exiting tunnels and underground parking lots). Thus, additional modules, possibly learning-based, are needed to pace λ estimation and to provide other system-wide inputs. Finally, it should be noted that system optimization with respect to computational hardware requirements is beyond the scope of this work. We have a high degree of confidence, however, that the solution can be efficiently implemented using novel mobile-oriented architectures, which have been at the focus of many research and development efforts in recent years [56,57].

## Figures and Tables

**Table 1:** Typical performance for different test configurations.

Deblurring Configuration	PSNR [dB]/SSIM
Initial: Tikhonov Deconvolution $\lambda =7.5\times 10^{-3}$	30.13 ± 3.04/0.926 ± 0.025
Initial + UW-64	34.11 ±3.14/0.964 ± 0.015
Initial + UW-128	34.46 ± 3.55/0.968 ± 0.016
Initial + residual U-Net	35.65 ± 3.95/0.974 ± 0.015

**Table 2:** Typical deblur quality before and after joint optimization.

Deblurring Configuration	PSNR [dB]/SSIM
Tikhonov Deconvolution $\lambda_{\mathrm{opt}}=7.5\times 10^{-3}$	30.13 ± 3.04/0.926 ± 0.024
Tikhonov Deconvolution $\lambda_{\mathrm{opt}}^{*}=3.3\times 10^{-3}$ (after joint training)	27.66 ± 1.46/0.818 ± 0.014
Initial + residual U-Net $\lambda_{\mathrm{opt}}=7.5\times 10^{-3}$	35.65 ± 3.95/0.974 ± 0.015
Initial + residual U-Net: Jointly Trained	36.90 ± 3.93/0.980 ± 0.012

**Table 3:** Mean PSNR/SSIM values for deblurring performance, before and after E2E training.

RegParamNet	RD before E2E	RD after E2E	IEN before E2E	IEN after E2E
Configuration	PSNR [dB]/SSIM	PSNR [dB]/SSIM	PSNR [dB]/SSIM	PSNR [dB]/SSIM
λ-Weights (T)	28.61±4.66/0.874±0.085	31.67 ± 4.35/0.76 ± 0.18	31.67 ± 4.34/0.932 ± 0.054	32.0 ± 5.0/0.94 ± 0.056
Regression (T)	28.58 ± 4.74/0.875 ± 0.09	28.0 ± 5.0/0.80 ± 0.14	32.84 ± 5.0/0.94 ± 0.053	33.0 ± 5.0/0.94 ± 0.054
λ-Weights (W)	25.16 ± 3.23/0.734 ± 0.107	25.17 ± 3.0/0.742 ± 0.10	27.49 ± 3.1/0.865 ± 0.064	27.54 ± 3.19/0.87 ± 0.064

**Table 4:** Comparison of the proposed method (NANBD) to state-of-the-art approaches. Best values are marked in bold.

Test	DBCNN [24]	MLP [50]	Son et al. [51]	NANBD	NANBD* (BDD Set)
Configuration	PSNR [dB]/SSIM	PSNR [dB]/SSIM	PSNR [dB]/SSIM	PSNR [dB]/SSIM	PSNR [dB]/SSIM
GaussianA	28.47/**0.8790**	27.16/0.8645	23.18/0.7347	**29.51**/0.8732	36.45/0.9744
GaussianB	25.34/0.7811	24.48/0.7766	22.88/0.6814	**29.14**/**0.865**	34.97/0.9608
GaussianC	22.79/0.7194	22.31/0.6752	22.17/0.659	**28.57**/**0.85**	33.62/0.95
GaussianD	-	-	-	**21.99**/**0.5477**	27.92/0.803
GaussianE	-	-	-	**25.14**/**0.7222**	29.99/0.8876
SquareA	22.90/0.7078	22.81/0.6975	17.74/0.4139	**28.57**/**0.8432**	34.99/0.9637
SquareB	24.01/0.7564	23.52/0.7375	19.29/0.4788	**28.91**/**0.8589**	34.45/0.956
SquareC	-	-	-	**21.52**/**0.7127**	29.99/0.8866
SquareD	-	-	-	**24.92**/**0.519**	27.61/0.8032
MotionA	27.93/0.8795	26.73/08448	27.15/0.8525	**30.65**/**0.8912**	36.27/0.9748
MotionB	25.50/0.8009	24.77/0.7726	24.49/0.7378	**29.34**/ **0.8819**	35.85/0.9716

## Data Availability

Not applicable.

## Supplementary Materials

The following supporting information can be downloaded at: https://www.mdpi.com/article/10.3390/s22186923/s1, Figures S1–S7: Additional examples of deblurred images for various blur kernels and noise intensities.

## Abbreviations

The following abbreviations are used in this manuscript:

1D/2D	One-Dimensional / Two-Dimensional
ADAS	Advanced Driver Assistance System(s)
AV	Autonomous Vehicle
BDD	Berkeley Deep Drive
BSD	Berkeley Segmentation Dataset
CNN/DNN	Convolutional Neural Network / Deep Neural Network
DSLR	Digital Single-Lens Reflex (camera)
E2E	End-to-End
FC	Fully Connected (layer)
GCV	Generalized Cross-Validation
ICM	Image Corruption Module
IEN	Image Enhancement Network
ISP	Image Signal Processing/Processor
MAP	Maximum A Posteriori (estimation)
MSE	Mean Squared Error
PSF	Point Spread Function
PSNR	Peak Signal-to-Noise Ratio
RD	Regularized Deconvolution
ReLU	Rectified Linear Unit
RMS	Root Mean Square
SGD	Stochastic Gradient Descent
SNR	Signal-to-Noise Ratio
SSIM	Structural Similarity
SVD	Singular Value Decomposition
UW	Uniform Width
