# Supplementary material for: Noise-Adaptive Non-Blind Image Deblurring
Source: Sensors (Basel). 2022 Sep 13;22(18):6923. doi: 10.3390/s22186923 (PMC9503865; doi:10.3390/s22186923)
Supplement: Supplementary file 1 [file sensors-22-06923-s001.zip › sensors-1884794-supplementary.pdf]

Supplementary Materials: Examples of Deblurred Images

- Below are several examples of noise-adaptive non-blind image deblurring as described in the main article (Section IV)
- Image quality has been tested in the following processing configurations:

a) Regularized Deconvolution output before E2E training
b) Regularized Deconvolution output after E2E training
c) Image Enhancement Network output before E2E training
d) Image Enhancement Network output after E2E training

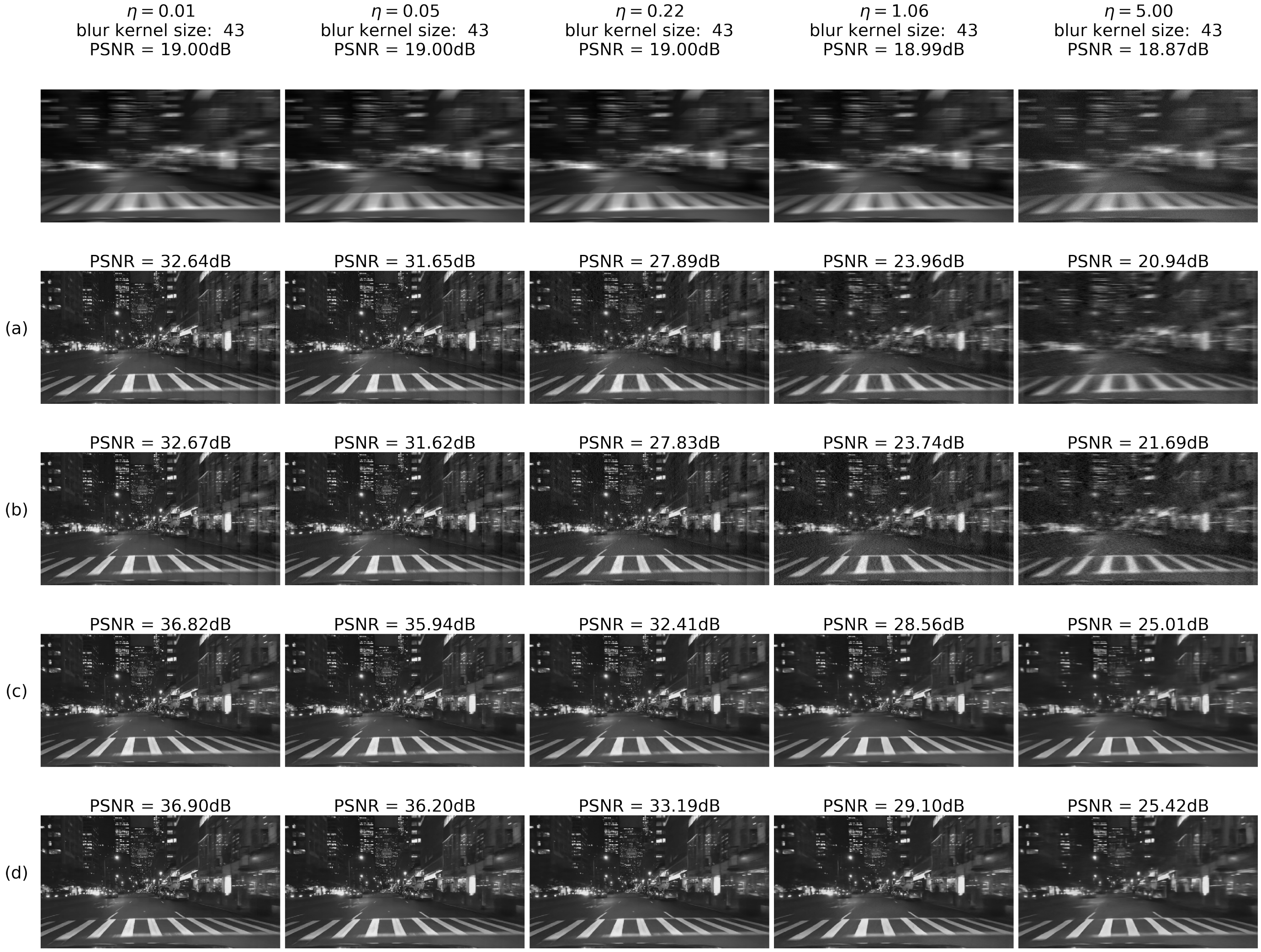

Figure S1. *Image I: Deblurring using Tikhonov deconvolution (RegParamNet: regression).*

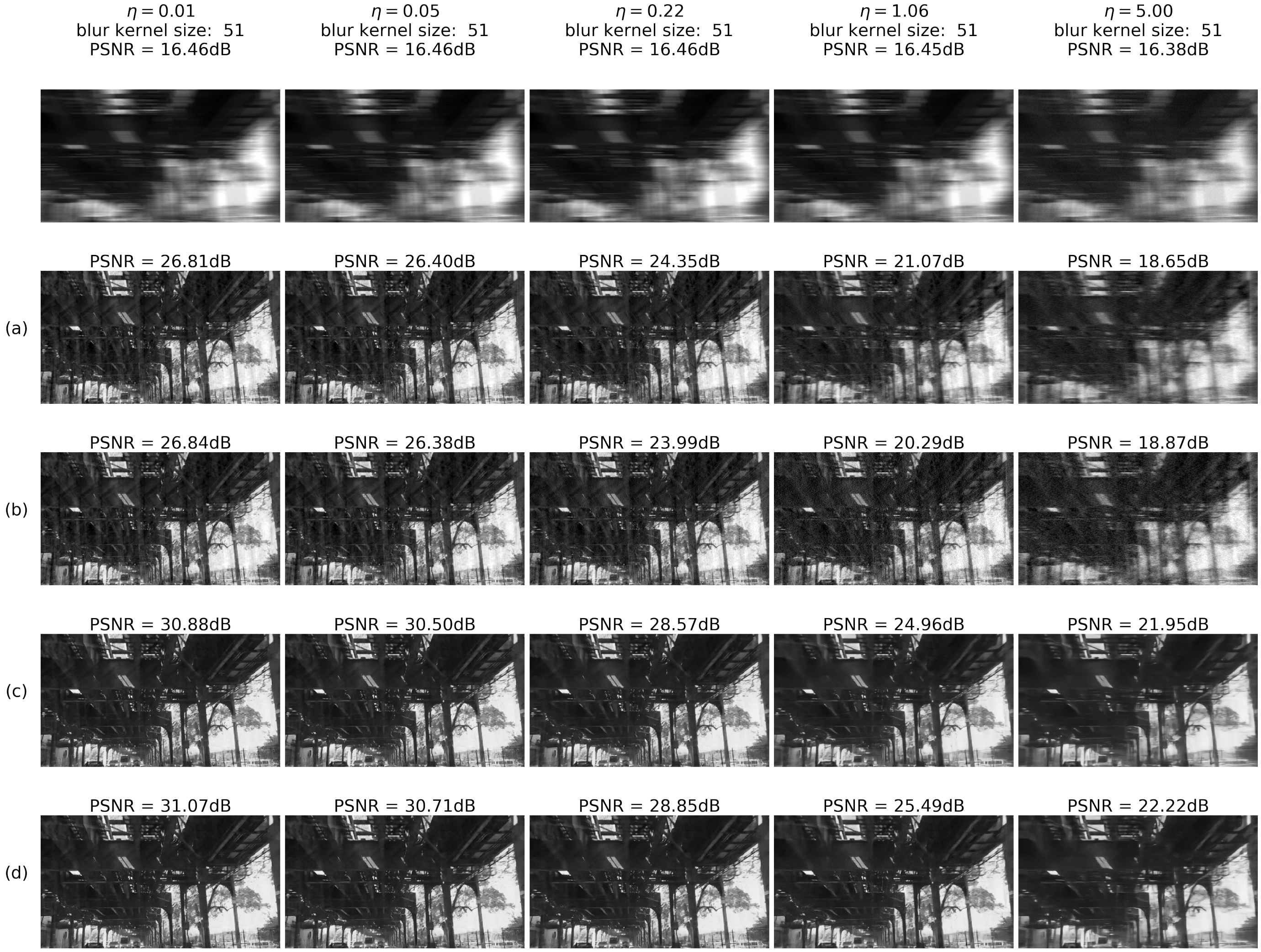

Figure S2. *Image II: Deblurring using Tikhonov deconvolution (RegParamNet: regression).*

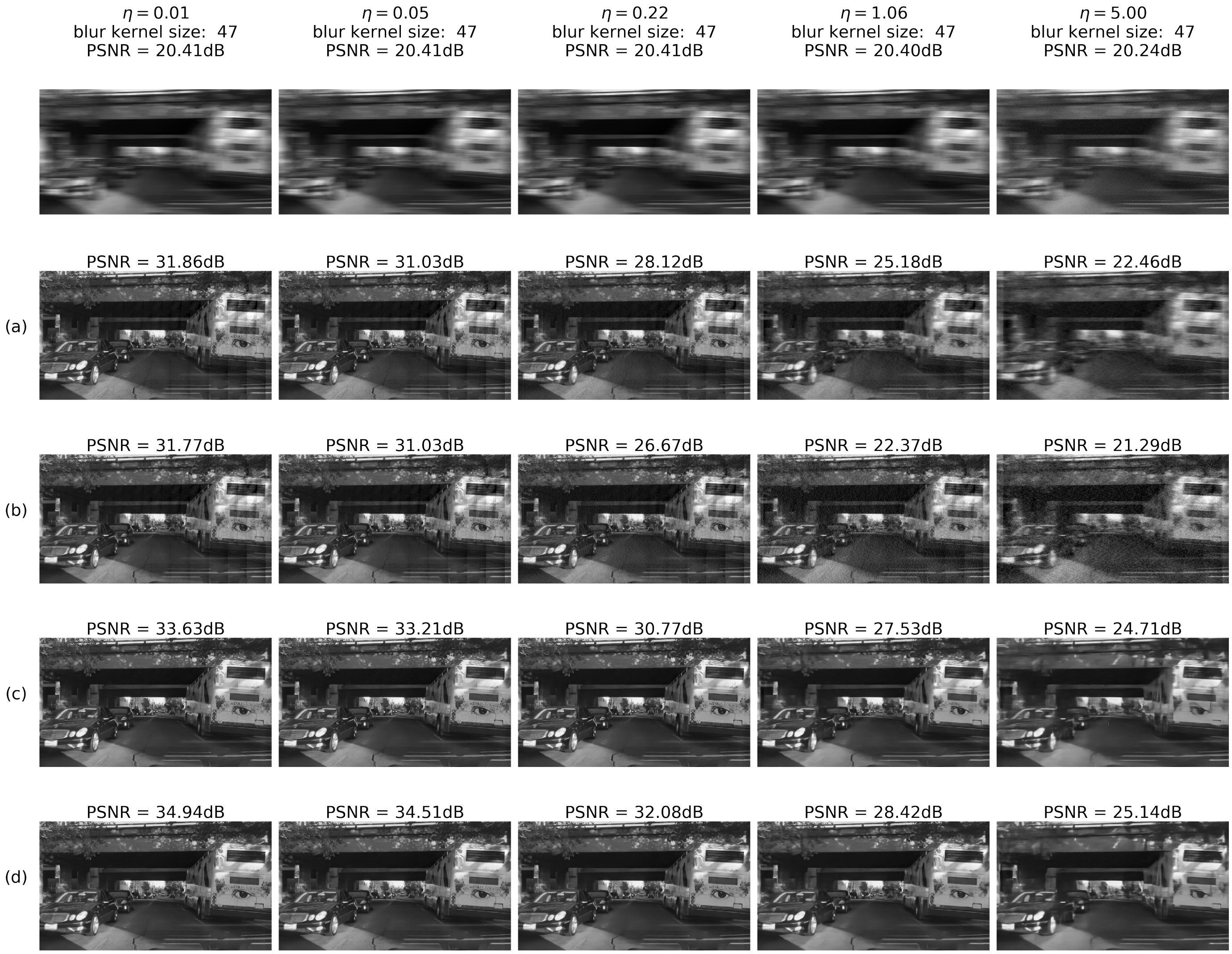

Figure S3. *Image III: Deblurring using Tikhonov deconvolution (RegParamNet: λ-weights).*

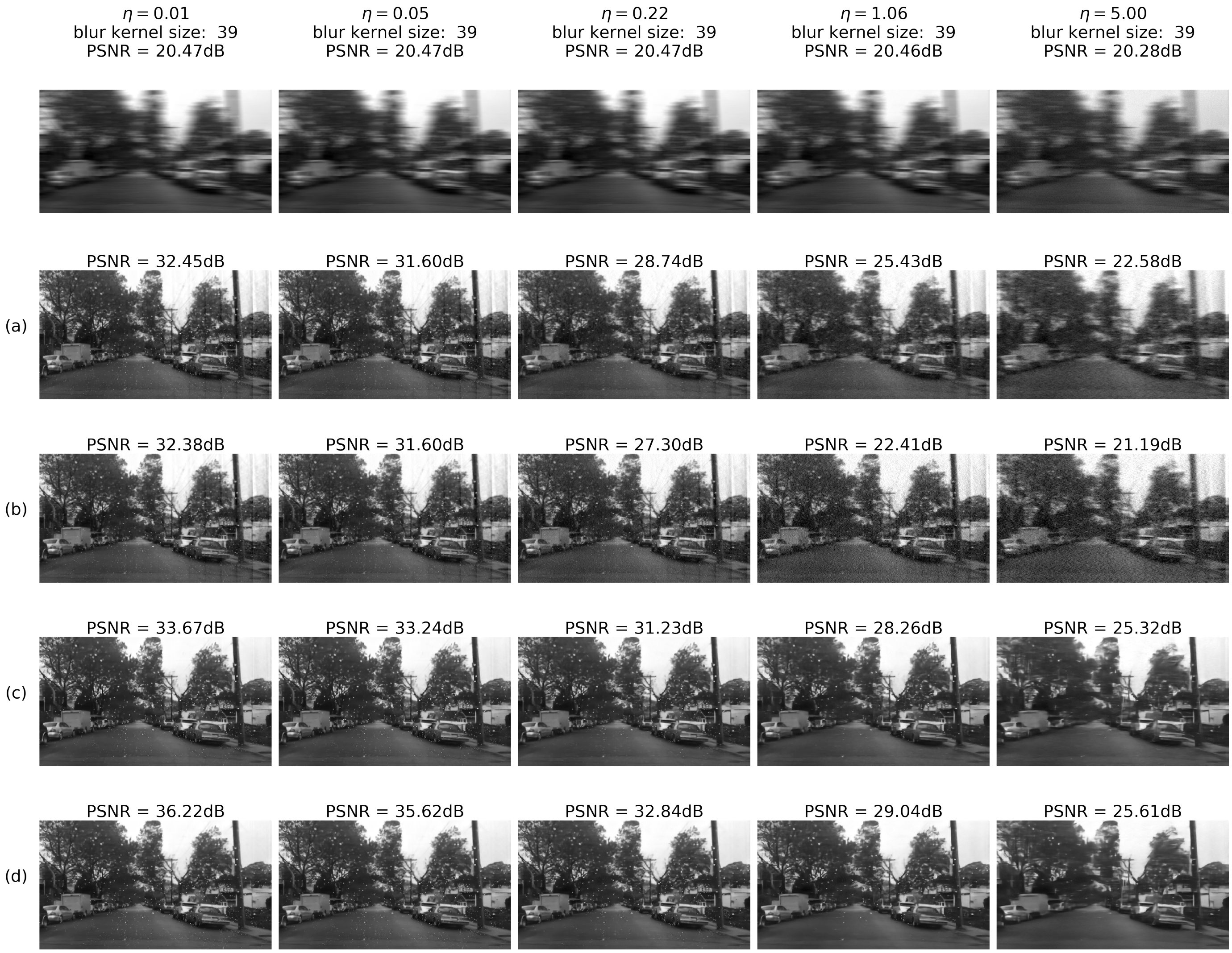

Figure S4. *Image IV: Deblurring using Tikhonov deconvolution (RegParamNet: λ-weights).*

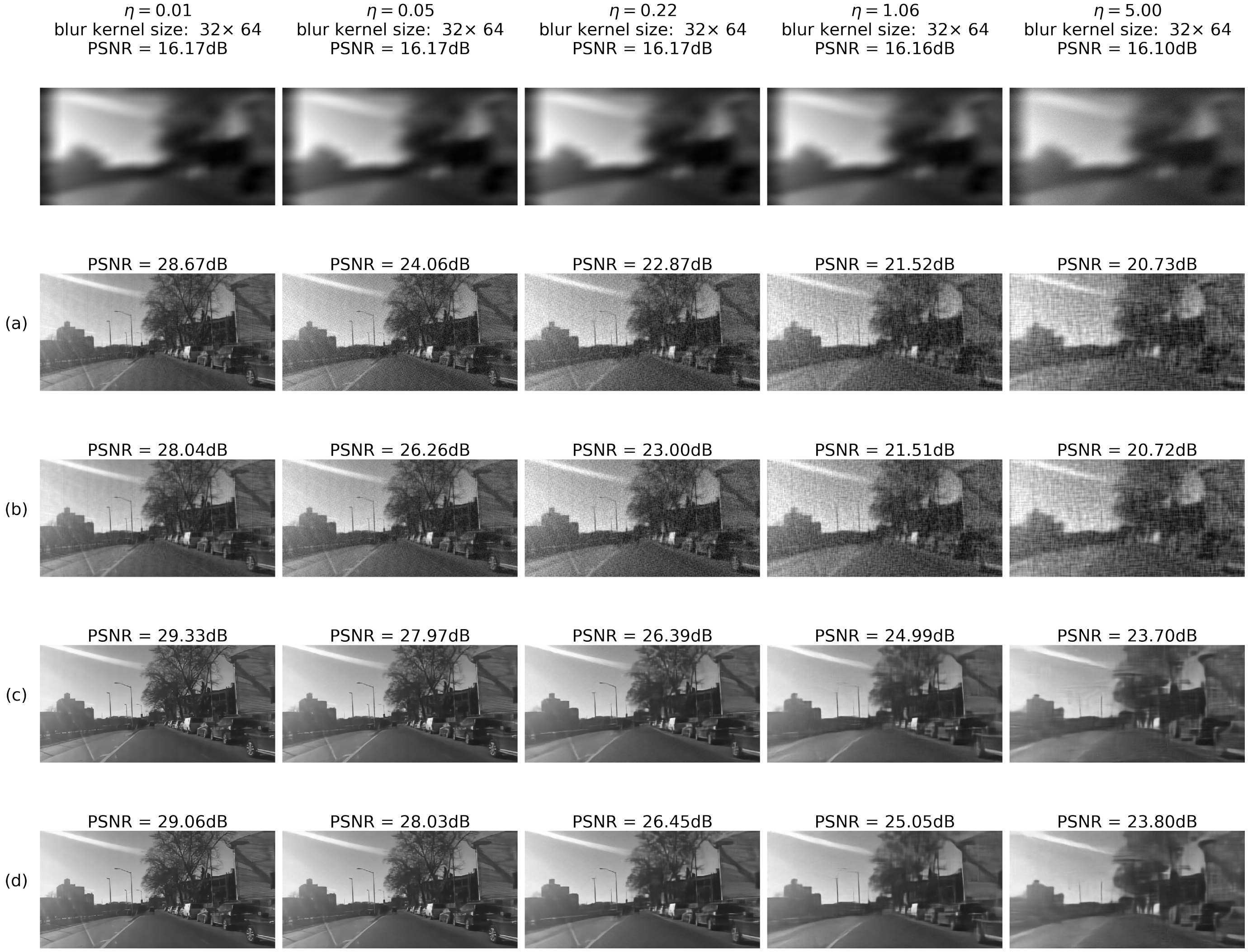

Figure S5. *Image V: Deblurring using Wiener deconvolution (RegParamNet: λ-weights).*

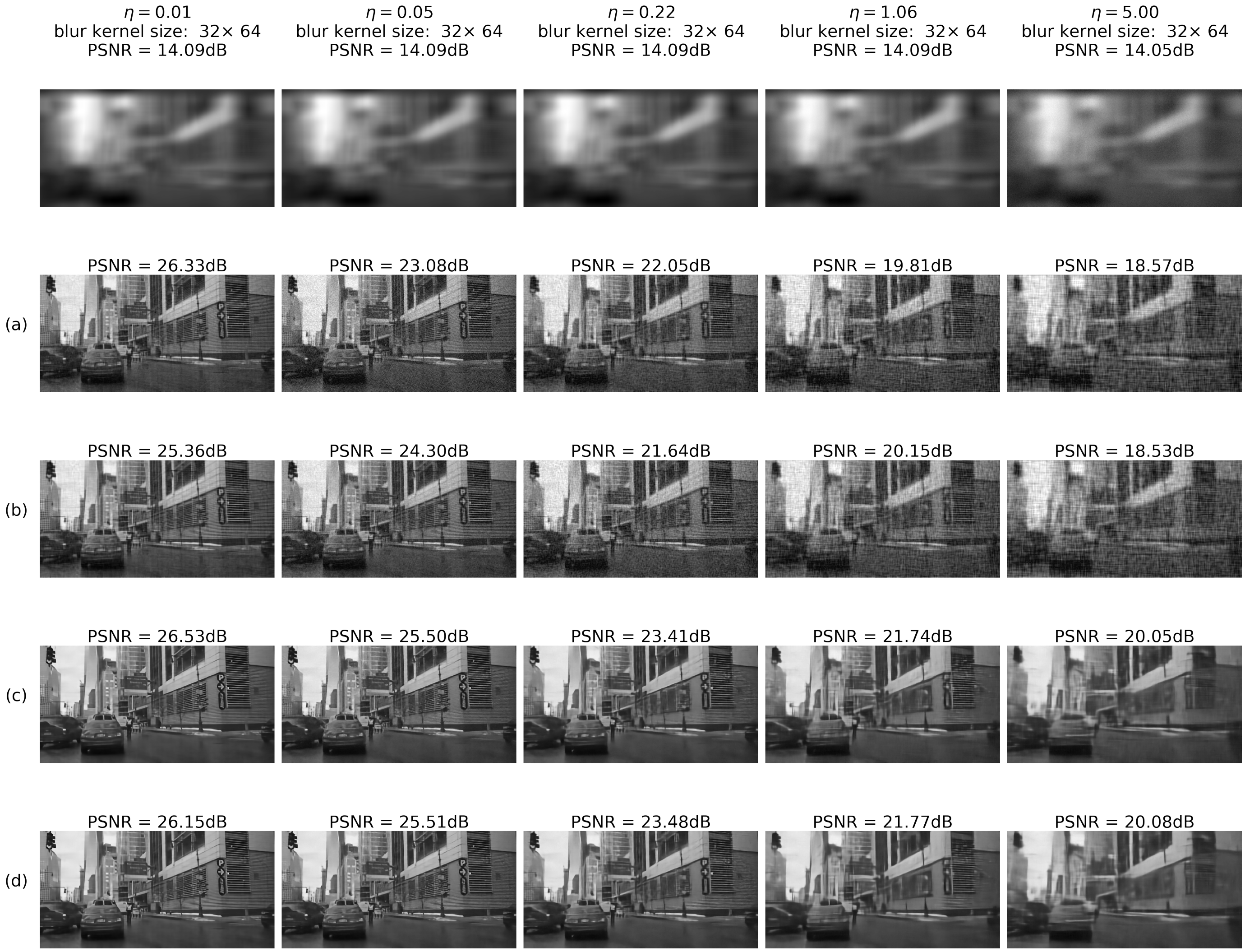

Figure S6. *Image VI: Deblurring using Wiener deconvolution (RegParamNet: λ-weights).*

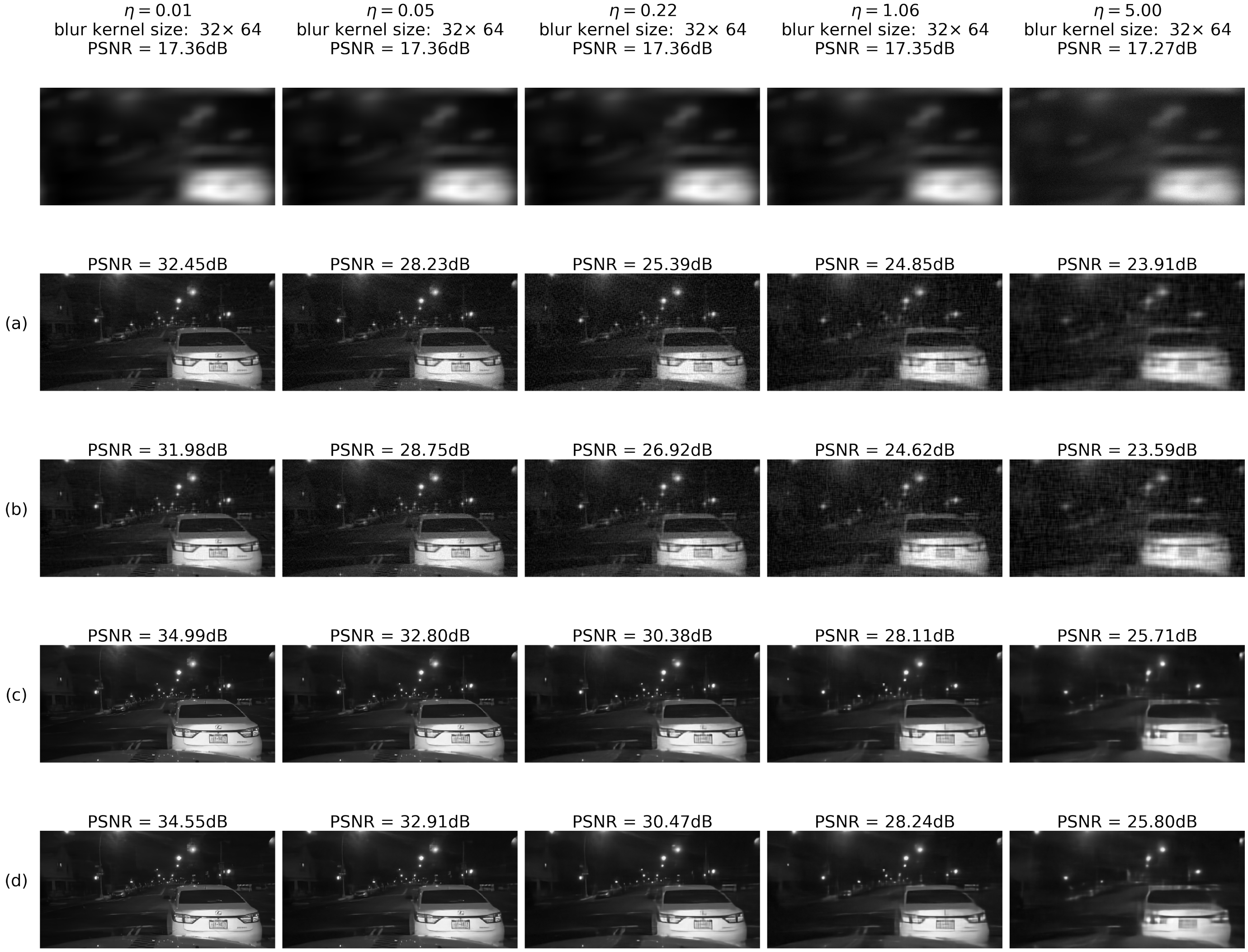

Figure S7. *Image VII: Deblurring using Wiener deconvolution (RegParamNet: λ-weights).*
